# A case of T-prolymphocytic leukemia harboring *RAS* mutation

**DOI:** 10.1007/s00277-026-06986-2

**Published:** 2026-04-09

**Authors:** Salvatore Perrone, Giada Pacitto, Alessia Tirnetta, Claudia Mulargia, Maria Rosaria Angelitti, Andrea Corbingi, Elettra Ortu La Barbera, Emiliano Fabiani, Maria Teresa Voso, Arianna Di Napoli

**Affiliations:** 1 Department of Hematology, S.M. Goretti Hospital, Polo Universitario Pontino, Latina, Italy; 2 Department of Translational Precision Medicine, University La Sapienza, Rome, Italy; 3 Division of Medical Genetics, Department of Experimental Medicine, San Camillo-Forlanini Hospital, Sapienza University, Rome, Italy; 4 Department of Biomedicine and Prevention, Tor Vergata University, Rome, Italy; 5 Department of Clinical and Molecular Medicine, School of Medicine and Psychology, Sapienza University, Sant’Andrea University Hospital, Rome, Italy

**Keywords:** T-prolymphocytic leukemia (T-PLL), Alemtuzumab, *KRAS*, Bendamustine

## Abstract

The text reports the clinical case of a 61-year-old woman with a long history of generalized pruritus who was eventually diagnosed with T-cell prolymphocytic leukemia (T-PLL), a rare and aggressive T-cell lymphoproliferative disorder. At presentation, the patient had fever, skin lesions, lymphocytosis, thrombocytopenia, coagulation abnormalities, and marked splenomegaly without lymphadenopathy. Morphological, immunophenotypic, and histological analyses of peripheral blood and bone marrow revealed diffuse infiltration by mature T lymphocytes with strong TCL1 expression, and T-cell receptor gene rearrangement confirmed clonality, supporting the diagnosis of T-PLL. The patient initially received bendamustine chemotherapy, which failed to induce a response. Second-line treatment with the anti-CD52 monoclonal antibody alemtuzumab was subsequently initiated, but the disease proved refractory, with persistent bone marrow involvement, worsening cytopenias, disease progression, and clinical deterioration. As a result, alemtuzumab was discontinued and the patient was transitioned to palliative care. A particularly notable finding was the detection, by next-generation sequencing, of a pathogenic KRAS G12A mutation, which has not previously been reported in T-PLL. This mutation was identified incidentally using a myeloid-targeted NGS panel. The finding broadens the known mutational spectrum of T-PLL and suggests that activation of the RAS signaling pathway may represent an alternative oncogenic mechanism beyond the canonical TCL1 and JAK–STAT pathways. We suggest that investigating for KRAS mutations in additional T-PLL cases may be beneficial to explore this pathogenetic pathway and could open new avenues for future targeted therapeutic strategies.

**Supplementary Information:**

The online version contains supplementary material available at 10.1007/s00277-026-06986-2.

## Introduction

T-PLL is a rare (2 per million per year) T-cell lymphoproliferative disorder characterized by a poor prognosis and an aggressive behavior [1]. The clinical presentation includes B-symptoms, hepato-splenomegaly, and usually marked lymphocytosis [2]. Here we present a case-report of a patient with T-PLL harboring a particular mutation that conferred further dismal outcome.

## Case presentation

In October 2024, a 61-year-old woman presented to the emergency department of our hospital with fever, generalized pruritus, and cutaneous lesions. She had a history of more than one year of pruritus, but several dermatological consultations were inconspicuous. Blood tests showed normal hemoglobin levels (11 g/dL), leukocytosis (13.0 × 10^9/L) with lymphocytosis (absolute lymphocyte count of 9.0 × 10^9/L), and thrombocytopenia (39 × 10 × 9/L). Elevated levels of serum lactate dehydrogenase (LDH, 500 U/L) and of bilirubin (BT (3.73MG/DL, BD 1.29 mg, BI 1.29) were detected. Uric acid and creatinine were within normal ranges. Coagulation tests showed: INR 2.1, fibrinogen 96 mg/dl, antithrombin III 50.6%, D-dimer 3.64 mg/dl.

A contrast-enhanced abdominal CT scan revealed splenomegaly (20 cm in maximum diameter) without lymphadenopathy. Examination of the peripheral blood smear showed monotonous, small- to medium-sized lymphoid cells (Supplemental Fig. 1). Peripheral blood flow cytometry analysis indicated an expanded population of lymphocytes, which accounted for 76% of viable WBC, with pathologic, mature T-cell immunophenotype.

Histopathological examination of the bone marrow biopsy revealed a diffuse infiltration by a monotonous lymphoid population positive for CD3, CD5, CD7, TCL1, CD4, and PD1, and negative for CD8, CD30, ALK1, TIA1, and TdT that accounted for 40% of the total cellularity. The residual hematopoietic cells showed normal maturation. These findings were consistent with involvement by a peripheral T-cell lymphoma, in which the strong expression of TCL1 suggested the diagnosis of T-cell prolymphocytic leukemia (T-PLL). Molecular clonality studies performed on peripheral blood showed a monoclonal rearrangement of the T-cell receptor gamma gene (TRG). Cytogenetics failed at diagnosis, and *TP53* was wild-type by sequencing. A skin biopsy to the right shoulder where the patient complained an itchy dyschromic lesion, showed a completely cleaved intradermal nevus and skin with epidermal hyperplasia.

The patient underwent a PET scan, which showed an enlarged spleen with a finely heterogeneous FDG distribution, maintaining a physiological hepato-splenic uptake ratio, and no evident focal areas of abnormal uptake. A mild and diffuse radiotracer uptake was also observed in the bone marrow.

She started chemotherapy consisting of four cycles of bendamustine. Disease reassessment at the end of the four chemotherapy cycles, performed with a contrast-enhanced CT scan, showed persistence of hepatosplenomegaly, lymphocytosis, and generalized pruritus. Blood tests revealed white blood cell (WBC) count of 6.8 × 10^9/L, with an absolute lymphocyte count of 5.73 × 10^9/L, a hemoglobin level of 7.3 g/dL, and a platelet count of 26 × 10 × 9/L. Serum lactate dehydrogenase (LDH) was elevated at 1000 U/L.

Following confirmation of persistent T-PLL on bone marrow biopsy, second-line therapy with (MoAb anti-CD52) alemtuzumab was initiated with progressively increasing doses, according to the following schedule: Day-1 3 mg, D-2 10 mg, D-3 30 mg, but further weekly doses of alemtuzumab were reduced at a dose of 15 mg due to thrombocytopenia.

Following the first cycle of therapy (1 month), the patient underwent a bone marrow aspiration showed heterogeneous cellularity averaging 60% and are the site of diffuse infiltration by a lymphoproliferative disease composed of medium-sized elements with a T-cell phenotype: CD3+, CD5+, CD7+, CD2+, with a proportion of CD4 + cells greater than CD8+, TCL1+, TdT–, CD1a–, LMO2–, CD34–, MPO–, CD25–, CD23–, CD10+/–, BCL6+/–, PD1+/–, BCL2+/– (weak), cyclin D1+, CD56–, TIA1–, GrzB–, ALK1–, CD79a–, CD20–, PAX5–, CD30–, EBER–, p53– (strong nuclear positivity in 4–5% of cells). The proliferative index (Ki67) was 20–30%. Occasional CD138 + plasma cells (2–5% of marrow cellularity), polytypic, were also present. The residual hematopoietic population was below 5%. Diffuse reticular fibrosis with focal formation of collagen bundles (MF-2) was present. The histological features observed indicated bone marrow involvement by peripheral T-cell lymphoma, consistent with the diagnosis of T-PLL.

**Fig. 1:**
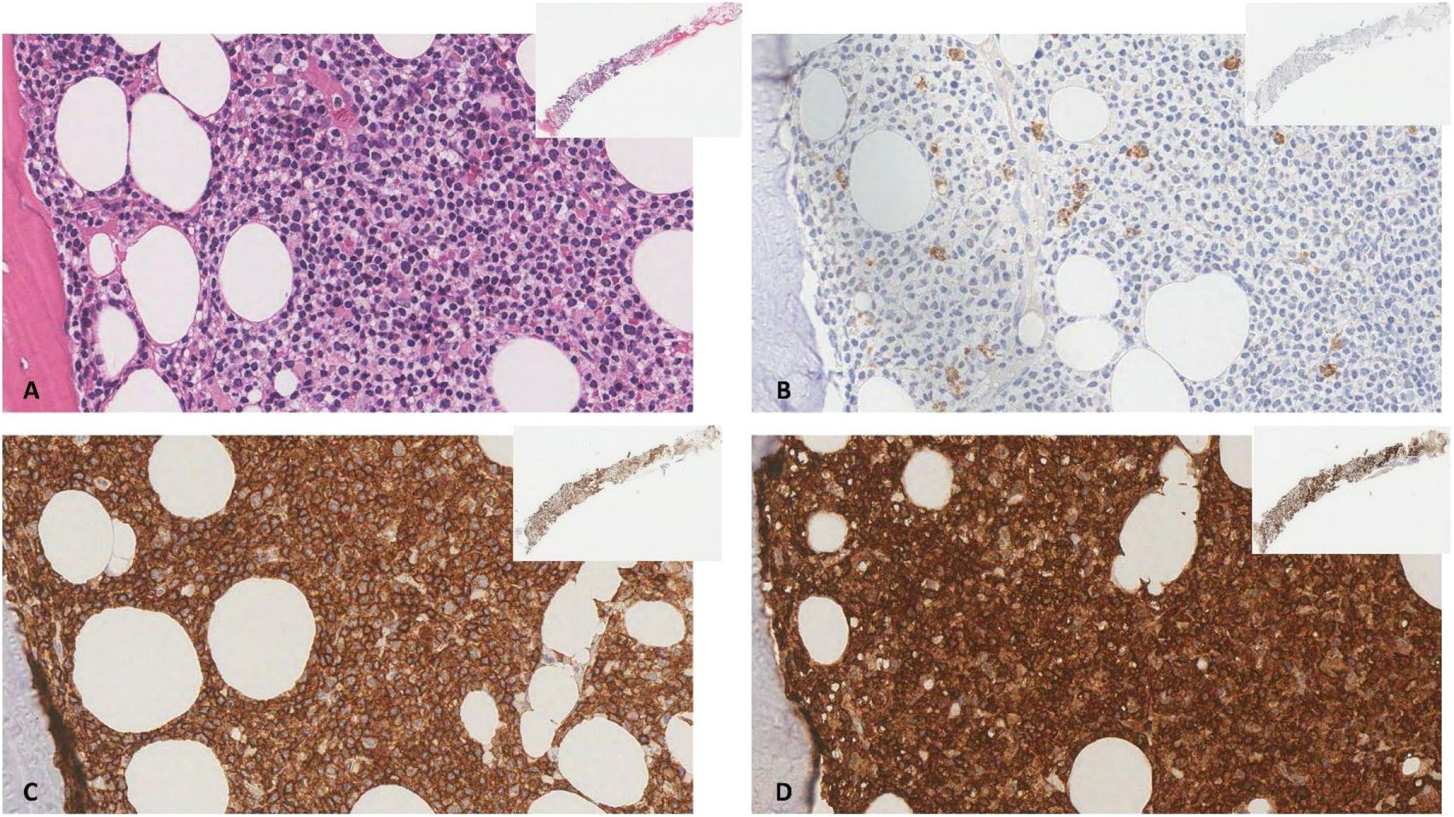
Bone marrow diffusely infiltrated by small-to-medium sized cells (**A**, original magnification (o.m.) x 400, insert o.m. x5), negative for MPO (**B**, o.m. x400, insert o.m. x5) but expressing CD3 (**C**, o.m. x 400, insert o.m. x5) and TCL1A (**D**, o.m. x 400, insert o.m. x5)

**Fig.2:**
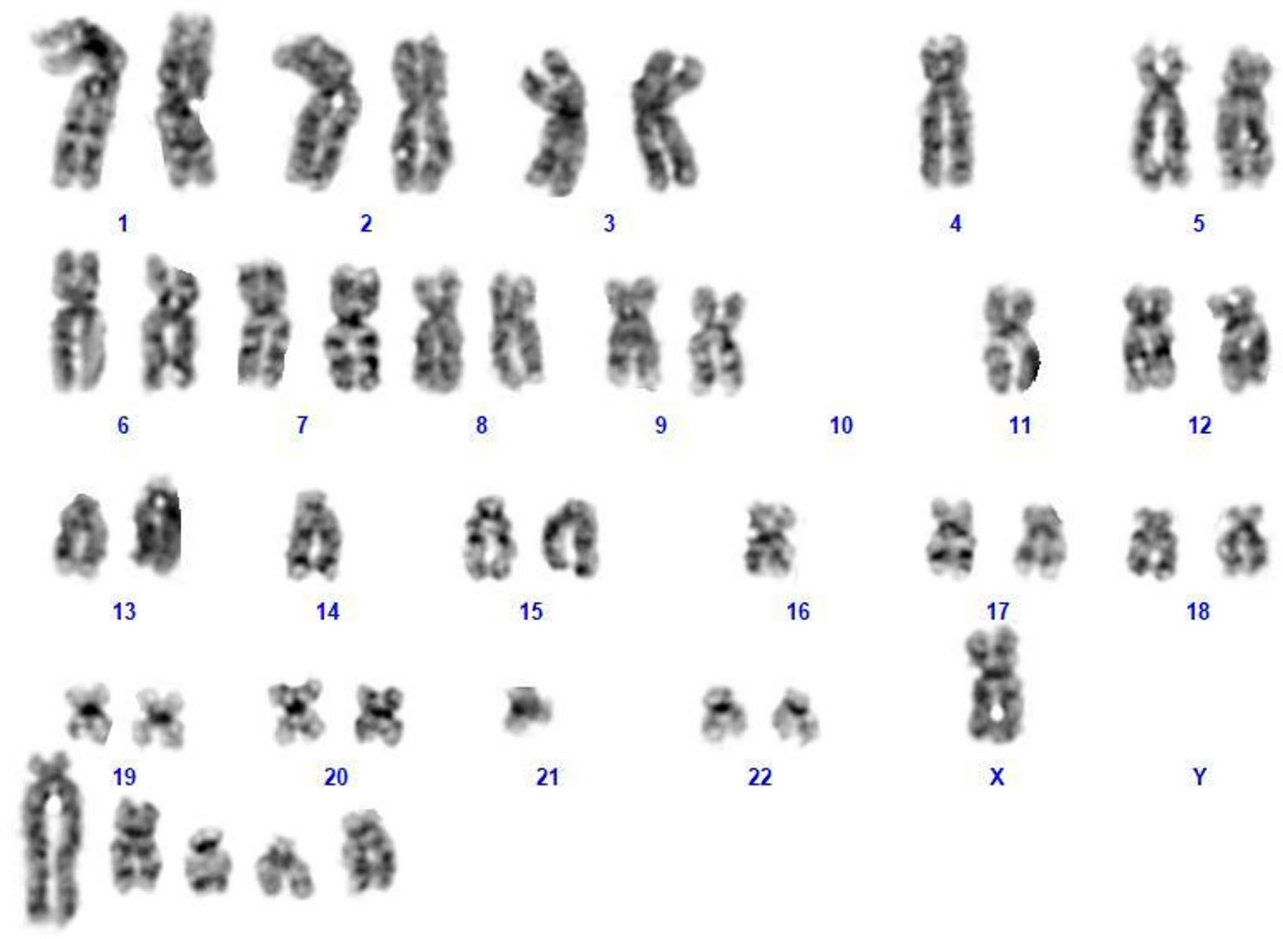
G-banding analysis demonstrated the presence of a complex karyotype with 43 chromosomes including five unidentified markers chromosomes due to the small number of cells (bone marrow sample)

Flow cytometry analysis confirmed persistence of 26.67% lymphocytes with the following immunophenotypic profile: CD10+ (8%), CD7+ (93%), CD5+ (93%), CD2+ (93%), CD3+ (93.29%), cytoplasmic CD3+ (98%), CD4+ (79%), and CD8+ (15%). The bone marrow biopsy (Fig. 1) showed 60% cellularity, with evidence of diffuse infiltration by small-to-medium-sized T-cells CD3+, CD5+, CD7+, CD2+, TCL1+, PD1+/-, with a predominance of CD4 + over CD8 + and p53 strong nuclear positivity in 4–5% of the cells. The cells were negative for TdT, CD1a, LMO2, CD34. The residual hematopoietic population was < 5%. Cytogenetic analysis of bone marrow blood revealed a complex karyotype no further characterized due to the small number of cells (Fig. 2). Next-generation sequencing (NGS) (targeted myeloid solution panel, 30 genes, Sophia Genetics) identified *KRAS* pathogenetic mutation c.35G > C (variant allele frequency (VAF) 38%).

In light of disease progression documented by bone marrow examination, the reappearance of lymphocytosis (10,000/mm³), lack of response to alemtuzumab, the development of pulmonary consolidation, and worsening of general clinical condition, it was decided to discontinue treatment with alemtuzumab. The patient was placed on palliative therapy.

## Discussion

The WHO 5th edition recognizes T-PLL with appropriate phenotype, T-cell monoclonality and the presence of genetic aberrations, including structural variants with breakpoints affecting the *TCL1A* or *MTCP1* locus or expression of TCL1 [3]. In particular, the rearrangements involving TCL1 (T-cell leukemia/lymphoma1) family genes TCL1A, MTCP1 (mature T-cell proliferation), or TCL1B (alias TCL1/MTCP1-like 1 [TML1]), are relatively specific for T-PLL and are present in more than 90% of cases, either as inv(14)(q11q32) or t(14;14)(q11;q32) (involving TCL1A or TCL1B), or t(X;14)(q28;q11) (involving MTCP1; mature T-cell proliferation). Unfortunately, in our case cytogenetic analysis was unable to identify these rearrangements due to the small number of cells, but TCL1a protein expression was identified by immunohistochemistry with a strong staining in the bone marrow lymphoid infiltrate [2]. The mutational landscape of T-PLL has been explored by several studies and alterations of the *JAK*-*STAT* pathway have been identified as the most frequent ones (75% of T-PLL) [4, 5]. Our patient showed a pathogenetic mutation of *KRAS*. To our knowledge this is the first report of *KRAS* mutation in T-PLL. We employed an NGS panel for myeloid neoplasms instead of a lymphoid-dedicated panel. Indeed, *RAS* mutations can be detected in AML and other myeloproliferative neoplasms [6–8]. In AML the presence of *RAS* mutations has also been implicated in resistance to targeted therapies, including FLT3 and IDH1/2 inhibitors [9, 10]. Moreover, *KRAS* mutations have been reported in other T-cell lymphomas, such as T-follicular helper (TFH)-derived peripheral T-cell lymphoma (PTCL) samples [11]. The identification of *KRAS* mutation in this patient could broaden the mutational spectrum of T-PLL. It could a hypothesis generating finding, that suggests that *RAS* pathway activation might represent an alternative oncogenic mechanism beyond the canonical *TCL1* and *JAK*-*STAT* pathways. Interestingly, in other solid neoplasms, like non-small cell lung cancer, can harbor *KRAS* mutations, and there are approved drugs targeting these genomic lesions [9]. Indeed, currently approved KRAS inhibitors, such as sotorasib and adagrasib, are selective for the *KRAS* G12C mutation (c.34G > T), but not for G12A or other non-G12C variants [12–15]. For tumors harboring *KRAS* G12A mutations, like our T-PLL case, management remains reliant on standard cytotoxic chemotherapy, immunotherapy, or other targeted agents based on tumor histology and molecular profile, but not on *KRAS*-specific inhibition [16]. Unfortunately, the poor conditions of our patient prevented us from associating alemtuzumab with chemotherapy (fludarabine, mitoxantrone, cyclophosphamide (FMC) or cladribine) [17, 18]. Conversely, the biology of our case was particularly unfavorable, since at least a transient response to alemtuzumab is generally seen in T-PLL [19]. In this single case, it is difficult to establish the sole biologic role of *KRAS* mutation, since other biological features could contribute (e.g., TCL1 expression [20]).

## Conclusion

In conclusion, this case emphasizes the opportunity of identifying *KRAS* in other patients with T-PLL. Since KRAS mutations directly impact the MAP-Kinase signaling pathway and have been previously identified in various PTCL subtypes, its presence in T-PLL may indicate an additional, albeit less frequent, pathogenetic driver within this aggressive malignancy. New therapeutic approaches are emerging for T-PLL, involving drugs that target autophagy, nuclear export, and inhibitor of apoptosis proteins (IAPs; birinapan) [21]. In this scenario, targeting *KRAS* might represent another option to be explored in the future.

## Supplementary Information

Below is the link to the electronic supplementary material.

Supplementary Material 1

## Data Availability

No datasets were generated or analysed during the current study.
