# Supplementary material for: A case of T-prolymphocytic leukemia harboring RAS mutation
Source: Ann Hematol. 2026 Apr 9;105(5):230. doi: 10.1007/s00277-026-06986-2 (PMC13065534; doi:10.1007/s00277-026-06986-2)
Supplement: Supplementary file 1 — Supplementary Material 1 [file 277_2026_6986_MOESM1_ESM.pdf]

Supplemental Figure 1

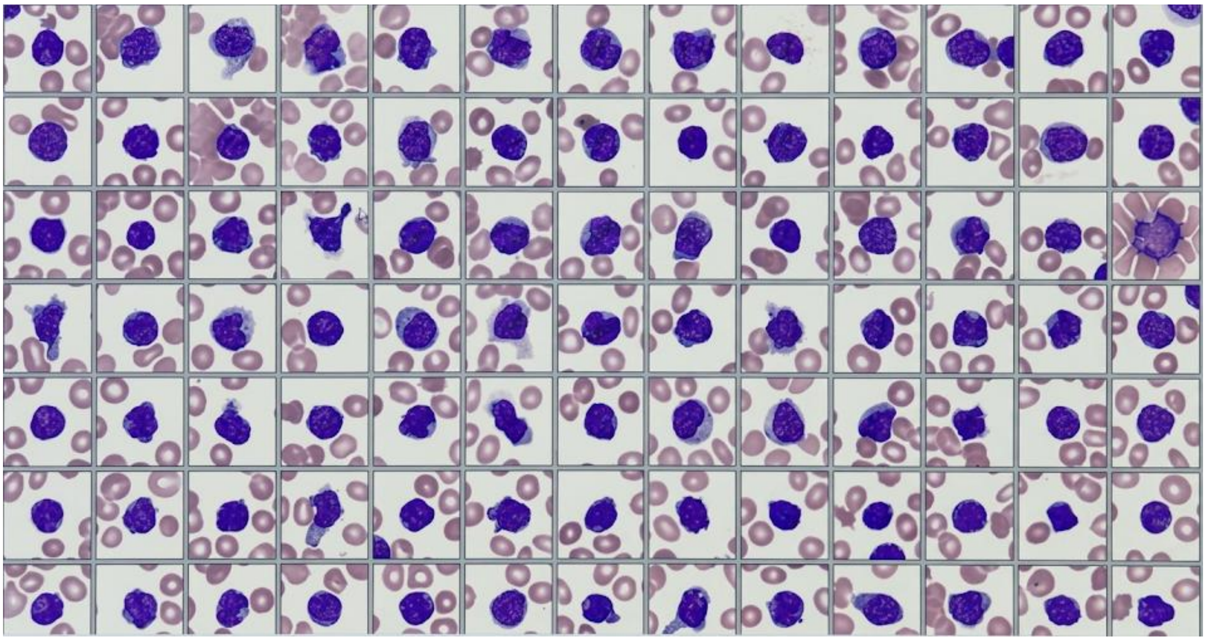

Atypical lymphocytes in peripheral blood stain. They are characterized by small- to- medium-size with scant cytoplasm and indented nuclei.
